# Extracorporeal membrane oxygenation in cardiovascular medication poisoning. A German-wide retrospective study

**DOI:** 10.1038/s41598-024-72547-0

**Published:** 2024-09-18

**Authors:** Benjamin Friedrichson, Thomas Jasny, Oliver Old, Florian Piekarski, Angelo Ippolito, Florian J. Raimann, Kai Zacharowski, Jan Andreas Kloka

**Affiliations:** 1 Department of Anaesthesiology, Intensive Care Medicine and Pain Therapy, University Hospital Frankfurt, Goethe University, Theodor-Stern-Kai 7, 60590 Frankfurt, Germany; 2 Department of Anesthesiology and Intensive Care Medicine, Rheinische Friedrich-Wilhelms-University, University Hospital Bonn, Bonn, Germany

**Keywords:** Cardiovascular medication poisoning, ECMO, Mortality, Nationwide, Arrhythmias, Heart failure, Outcomes research, Cardiac device therapy, Interventional cardiology

## Abstract

Medication poisoning, resulting from the ingestion of cardiotoxic drugs, presents a significant health issue. The mortality rate remains high for patients with myocardial dysfunction refractory to conventional treatments. Venoarterial Extracorporeal Membrane Oxygenation (V-A ECMO) provides temporary support, potentially enhancing patient outcomes. This study aims to assess the efficacy of V-A ECMO in treating cardiovascular failure induced by cardiovascular medication poisoning. We utilized inpatient data from all hospitalisations in Germany from 2007 to 2022 due to cardiovascular medication poisoning treated with V-A ECMO. Patient characteristics, comorbidities, complications and application of ECMO were described descriptively and analysed for statistical significance between survivors and non-survivors. Overall, 49 patients received V-A ECMO for cardiovascular medication poisoning, with a survival rate of 63.6%. The most ingested medications were calcium-channel blockers (38.8%) and beta-adrenoceptor antagonists (34.7%). Half of non-survivors received in-hospital CPR, compared to 12.9% of survivors. Early ECMO implantation (within 24 h of admission) was common (83.7%) but did not significantly impact survival rates. A substantial number of patients presented with multiple substances ingested. V-A ECMO represents a viable option for patients experiencing cardiac failure due to medication poisoning. A structured implementation of V-A ECMO for cardiovascular medication poisoning could lead to higher survival rates.

## Introduction

Patients presenting with intoxication exhibit a diverse range of symptoms, contingent upon the type and dosage of the ingested drug. In severe instances, poisoning can result in drug-induced cardiovascular failure, particularly when cardiotoxic substances are involved^1^. Some substances have been demonstrated to induce both cardiac and pulmonary failure^2,3^. Extracorporeal membrane oxygenation (ECMO) has emerged as a vital tool in today’s intensive care medicine practices, indicated for both cardiovascular and pulmonary failure^4,5^.

Medication poisoning can occur either accidentally or intentionally. In 2005, over 10,000 deaths in Europe were attributed to accidental poisoning^6^. Globally, approximately 140,000 individuals die annually from intentional medication poisoning^7^. Although cardiovascular drugs account for only 4% of toxic substance exposures, they account for 13% of resulting fatalities^8^. Given that most patients poisoned by cardiovascular drugs experience myocardial dysfunction, the use of venoarterial (V-A) ECMO is rational, as the poisoning is typically transient. Despite limited evidence, the latest ERC guideline recommends consideration of ECMO for poisoning with certain medication, including cardiovascular drugs^9^.

Given the high incidence of life-threatening intoxications, there is an urgent need for studies to gain knowledge on the application of V-A ECMO treatment in patients suffering from poisoning—especially due to cardiovascular medication. This study aims to investigate the utilization of V-A ECMO for patients experiencing cardiac failure due to cardiovascular medication poisoning.

## Methods

This study utilized inpatient data from all hospitalisations in Germany from 2007 to 2022. Due to regulatory constraints, only data up to 2022 were accessible at the time of the analysis. The data were provided by the Federal Statistical Office of Germany^10^. All hospitals in Germany are legally mandated to report every inpatient case, including diagnoses coded according to the International Statistical Classification of Diseases and Related Health Problems (ICD) and performed procedures according to process keys (‘*Operationen- und Prozedurenschlüssel*’—OPS).

To include patients with poisoning caused by medication that interferes with cardiovascular function, medications primarily affecting the autonomic nervous system (ICD T44.-) and medications primarily affecting the cardiovascular system (ICD T46.-) were selected for consideration (Table 1). All patients received V-A ECMO (OPS 8–852.3). All age groups were included.

**Table 1:** ICD codes used as inclusion criteria for medication poisoning.

ICD code	Definition
T44.-	Drugs primarily affecting the autonomic nervous system
T44.0	Anticholinesterase agents
T44.1	Other parasympathomimetics [cholinergics]
T44.2	Ganglionic blocking drugs
T44.3	Other parasympatholytics [anticholinergics and antimuscarinics] and spasmolytics
T44.4	Predominantly alpha-adrenoreceptor agonists
T44.5	Predominantly beta-adrenoreceptor agonists
T44.6	Alpha-adrenoreceptor antagonists
T44.7	Beta-adrenoreceptor antagonists
T44.8	Centrally acting and adrenergic-neuron-blocking agents
T44.9	Other and unspecified drugs primarily affecting the autonomic nervous system
T46.-	Agents primarily affecting the cardiovascular system
T46.0	Cardiac-stimulant glycosides and drugs of similar action
T46.1	Calcium-channel blockers
T46.2	Other antidysrhythmic drugs, not elsewhere classified
T46.3	Coronary vasodilators
T46.4	Angiotensin-converting-enzyme inhibitors
T46.5	Other antihypertensive drugs, not elsewhere classified
T46.6	Antihyperlipidaemic and antiarteriosclerotic drugs
T46.7	Peripheral vasodilators
T46.8	Antivaricose drugs, including sclerosing agents
T46.9	Other and unspecified agents primarily affecting the cardiovascular system

Data were analysed descriptively. Hospital mortality was calculated for all patients receiving V-A ECMO due to cardiovascular medication poisoning. Predefined comorbidities, complications and the Elixhauser score—a metric capturing the presence and severity of coexisting conditions in relation to hospital mortality based on administrative data—were examined for all patients and analysed for statistically significant differences between survivors and non-survivors^11,12^. For survivors, discharge destinations were examined after categorising into the categories “regular discharge”, “discharge into a rehabilitation facility”, “transfer to another hospital”, “discharge into a long-term care facility” and “discharge into a hospice”. The main indications for hospitalisation and secondary diagnoses were analysed by frequency of coding. Timestamps for V-A ECMO implantation were used to determine the number of hours between hospital admission and initiation of ECMO support. Early ECMO implantation was defined as occurring within 24 h of admission.

Group differences were examined utilizing the Chi-square test for binary variables and the Wilcoxon-rank sum test for continuous variables, as none of the considered variables were normally distributed. SAS (Version 9.4M6, SAS Institute Inc., Cary, NC, USA) was used for statistical analysis.

Due to institutional anonymisation, no conclusions about individual patients can be drawn from the data. According to §21KHEntgG, reimbursement data are free for scientific use. The Ethics Committee of the University Hospital Frankfurt waived the need for Ethics Committee approval for this study and informed consent (Chair: Prof Dr Harder, Ref. 2022-766). All data processing was performed according to the Declaration of Helsinki.

## Results

**Fig. 1:**
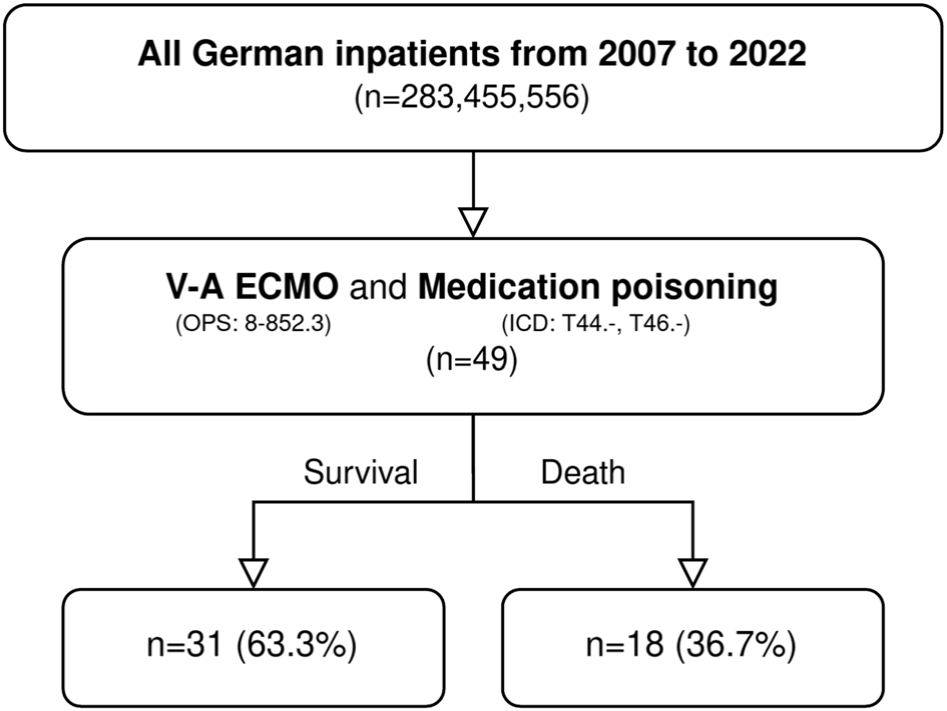
Patient flowchart.

In total, 49 patients were hospitalised due to cardiovascular medication poisoning and received ECMO between January 1st 2007 and December 31st 2022 (Fig. 1). Rate of survival was 63.3% with 31 survivors and 18 non-survivors.

### Demographics

Female patients constituted 57.1% (n = 28) of all patients. There was no significant difference (p = 0.30) in gender distribution between survivors and non-survivors, with 16 survivors (51.6%) and 12 non-survivors (66.7%) being female. Overall, the median (Interquartile range (IQR)) age was 54 years (38; 61). The median age did not significantly differ between survivors, with an age of 53 years (32; 61), and non-survivors, with an age of 56.5 years (45; 61). The median Elixhauser score was 13 (5; 20) overall. There was no significant difference between survivors and non-survivors, with a median of 13 (5; 20) for survivors and a median of 14 (9; 20) for non-survivors (Table 2).

**Table 2:** Patient characteristics.

	Total	Survivors	Non-survivors	p value
Total patients, n	49	31	18	
Female, n (%)	28 (57.1%)	16 (51.6%)	12 (66.7%)	0.30
Age (year), median (Q1; Q3)	54 (38; 61)	53 (32; 61)	56.5 (45; 61)	0.25
Elixhauser score, median (Q1; Q3)	13 (5; 20)	13 (5; 21)	14 (9; 20)	0.55
Hospital stay (d), median (Q1; Q3)	11.5 (5.1; 25.6)	14.6 (10.5; 42.2)	3.1 (1.4; 6.2)	< 0.01
Ventilated patients, n (%)	46 (93.9%)	28 (90.3%)	18 (100.0%)	0.17
Comorbidites, n (%)				
Depression	28 (57.1%)	19 (61.3%)	9 (50.0%)	0.44
Congestive heart failure	21 (42.9%)	14 (45.2%)	7 (38.9%)	0.67
Hypertension	19 (38.8%)	12 (38.7%)	7 (38.9%)	0.99
Complications, n (%)				
CPR after hospitalisation	13 (26.5%)	4 (12.9%)	9 (50.0%)	< 0.01
Asystole	12 (24.5%)	3 (9.7%)	9 (50.0%)	< 0.01
Anoxic brain damage	4 (8.2%)	*	*	0.10
Dialysis requirement	27 (55.1%)	14 (45.2%)	13 (72.2%)	0.07
ECMO duration (h), n (%)				
< 48	18 (36.7%)	*	*	
48–96	12 (24.5%)	*	*	
96–144	9 (18.4%)	*	*	
144–192	3 (6.1%)	*	*	
> 192	7 (14.3%)	*	*	
Discharge destination, n (%)				
Regular discharge	7 (22.6%)			
Rehabilitation facility	5 (16.1%)			
Transfer to another hospital	19 (61.3%)			

*CPR* cardiopulmonary resuscitation.

Characteristics marked with * are censored due to institutional data privacy guidelines.

All analysed comorbidities showed statistically insignificant differences. Depression was the most frequently coded comorbidity, with 19 patients (61.3%) in the survivor group and nine patients (50.0%) in the non-survivor group.

Cardiopulmonary resuscitation (CPR) was found to be statistically insignificant between survivors and non-survivors when performed prior to hospitalisation but statistically significant when performed after hospitalisation (p < 0.01). Ventricular fibrillation and flutter, and pulseless electrical activity did not differ significantly. Asystole was statistically significant, with three patients (9.7%) in the survivor group and nine patients (50.0%) in the non-survivor group (p < 0.01). Dialysis requirement was not significantly associated with survival.

The leading group of ingested medications was calcium-channel blockers (T46.1), coded 19 times (38.8%), followed by beta-adrenoreceptor antagonists (T44.7), coded 17 times coded (34.7%). Overall, poisoning due to drugs primarily affecting the autonomic nervous system (T44.-) was coded 23 times (46.9%) and poisoning due to agents primarily affecting the cardiovascular system (T46.-) was coded 42 times (85.7%) (Table 3).

**Table 3:** Ingested medication divided by coding.

Medication	n	%
Agents primarily affecting the cardiovascular system (T46.-)	42	85.7
Calcium-channel blockers (T46.1)	19	38.8
Other antihypertensive drugs (T46.5)	8	16.3
Angiotensin-converting-enzyme inhibitors (T46.4)	5	10.2
Cardiac-stimulant glycosides and drugs of similar action (T46.0)	4	8.2
Other antidysrhythmic drugs (T46.2)	4	8.2
*	2	4.1
Drugs primarily affecting the autonomic nervous system (T44.-)	23	46.9
Beta-adrenoreceptor antagonists (T44.7)	17	34.7
Other parasympatholytics [anticholinergics and antimuscarinics] and spasmolytics (T44.3)	5	10.2
*	1	2.0

Classes marked with * are censored due to institutional data privacy guidelines. Classes not represented in the population are not listed.

### ECMO usage

In 41 patients (83.7%) V-A ECMO was implanted within the first 24 h (early implantation). There was no statistically significant difference in survival between early and late ECMO implantation. The number of survivors and non-survivors receiving early and late ECMO had to be censored due to institutional data privacy guidelines, although statistical analysis was possible.

Three patients (9.7%) in the survivor group were not ventilated, whereas all non-survivors received mechanical ventilation. There was no statistically significant difference.

### Coded ICD-diagnoses

“Cardiogenic shock” and “acute respiratory insufficiency” were the most frequently coded diagnoses, each with 35 codings (71.4%). “Hospital-acquired pneumonia” was coded 14 times (28.6%), and “*hypotension due to drugs*” was coded 8 times (16.3%).

### Discharge destinations

Out of all 31 survivors, seven patients (22.6%) were discharged home, five patients (16.1%) were discharged to rehabilitation facilities, and 19 patients (61.3%) were transferred to another hospital.

## Discussion

There is a strong rationale for the use of V-A ECMO in patients experiencing poisoning due to cardiovascular medication, as the myocardial dysfunction is typically transient. While antidotes against cardiotoxics have improved the patients’ prognoses, mortality in poisoned patients can reach 90% when myocardial dysfunction is refractory to conventional treatments^13,14^.

With a survival rate of 63.6%, our study aligns with previous research. A retrospective analysis by Weiner et al., using the Extracorporeal Life Support Organizations (ELSO) registry, found a slightly lower survival rate with 52.7% for patients intoxicated with cardiovascular medication^15^. Ramanathan et al. used the same registry and reported a survival rate of 59% in adult patients with poisoning supported by V-A ECMO^16^. It should be emphasised that the populations in both studies were significantly younger than in the current study while reporting a lower survival rate. Weiner et al.^15^ reported a median age of 35 years (27; 52), while Ramanathan et al.^16^ reported a slightly lower age. The median age in the present study was 54 years (38; 61). Both studies, as well as the current study, found no statistically significant differences when comparing survivors and non survivors, indicating that ECMO support is also viable for older patients. It is worth considering that ELSO registry data are voluntarily submitted by specialised ECMO centres, which can lead to expertise bias. Unlike these analyses, the present study leverages a comprehensive, real-world dataset from across Germany, where data submission is mandated for all hospitals, regardless of their expertise, reflecting a broader spectrum of clinical practice. Our previous publications^17,18^, using this national dataset for ARDS patients on ECMO and for COVID-19 patients, demonstrated significantly higher mortality rates than those reported in the ELSO registry. A similar discrepancy was observed when analysing ECMO use in drowned patients^19^. This inconsistency underscores the importance of comprehensive mandatory reporting datasets to accurately reflect survival rates, revealing that high survival rates are achievable even outside specialized centres and across different age groups, thus providing a more realistic overview of ECMO effectiveness in poisoning cases across the country.

Overall, data on poisoning treated with ECMO is rare and mostly confined to single-centre experiences. The available literature shows a wide range of survival rates, from 14 to 75%^20–25^. Due to small study sizes and discrepancies in the types of ingested medications, comparisons are challenging.

This study revealed a statistically significant difference in in-hospital CPR between survivors and non-survivors. While only 12.9% of survivors received in-hospital CPR, 50.0% of non-survivors received in-hospital CPR. Whether this is due to a higher severity of cardiovascular poisoning in the non-survivor group or due to delayed ECMO implantation is unknown. Since both groups required ECMO support as a rescue therapy, it is reasonable to assume similar severity of poisoning. Unfortunately, research on this topic is currently lacking.

It is surprising that three patients (6.1%) were not ventilated. Awake ECMO is an alternative to invasive mechanical ventilation in non-intubated, spontaneously breathing patients^26^. The rationale for this type of ECMO usage is that it avoids several side effects related to the intubation and mechanical ventilation, as well as sedation. Clinical data on this topic and awake ECMO in general is scarce and mostly pertains to patients experiencing ARDS^26–28^. Considering that this study analysed a large timespan and awake ECMO is a novel practice, we are surprised to see that more than 6% of all patients were awake during ECMO support. Sedation in poisoned patients who possibly ingested multiple substance classes, or where ingested substance classes are not initially known, can be especially challenging. Awake patients can aid physicians by providing information on ingested medications and symptoms, and also lessen the need for sedation, provided they are cooperative. Especially in cases of medication poisoning, this might be less common due to higher incidences of complications, such as drug-induced psychosis. With no clear recommendation on awake ECMO, we did not expect to find this practice in a specialised patient population with low case volume. Unfortunately, closer analysis of these patients was not possible due to their low number and institutional privacy guidelines.

With agents primarily affecting the cardiovascular system coded 42 times (85.7%) and drugs primarily affecting the autonomous nervous system coded 23 times (46.9%), it is evident that some patients ingested multiple substance classes, possibly indicate non-accidental poisoning or poisoning with suicidal intent. This theory is supported by the frequent coding of depression as a comorbidity (n = 28, 57.1%). Substances most commonly used with suicidal intent are sedative-hypnotic drugs^29–31^. These substances, especially benzodiazepines, account for a large number of attempted and successful suicide cases^1^. A concern with intoxications involving benzodiazepines is respiratory depression or even respiratory insufficiency. “Acute respiratory insufficiency” was the most frequently coded diagnosis alongside “cardiogenic shock” (n = 35, 71.4%). One possible reason could be simultaneous ingestion of benzodiazepines. Unfortunately, the nature of the dataset does not allow for distinction between diagnoses on initial patient presentation and those developed during the patient’s hospital stay. Therefore, we cannot conclusively determine that respiratory insufficiency was a direct result of medication ingestion. Considering that “hospital-acquired pneumonia” was coded 14 times (28.6%), it is reasonable to assume that many patients presenting with respiratory insufficiency may have developed this condition during their hospital stay. However, with 41 patients (83.7%) having received ECMO implantation within the first 24 h after hospital admission, we can be reasonably certain that most patients received ECMO due to cardiovascular poisoning and not due to hospital-acquired complications. While most observations included the coding “cardiogenic shock” (71.4%), “hypotension due to drugs” was coded in 16.3% of patients. Whether cardiogenic shock was followed by hypotension, or hypotension was followed by cardiogenic shock is unclear. However, it is reasonable to assume that some patients received ECMO implantation primarily due to hypotension, especially since the leading group of ingested medication, “calcium-channel blockers”, are vasoactive.

Poisoned patients with suicidal intent often present with ingestion of multiple substances, significantly impacting mortality. They may also exhibit symptoms not directly related to the poisoning itself. An attempted suicide by medication overdose, classified as a “soft suicide”, may be accompanied by forms of “hard suicide”. These issues are rarely addressed in the literature but are common, as evidenced by more than half the patients in our study population presenting with depression. Evaluating the effectiveness of ECMO in poisoned patients might be biased due to these problems; however, the data accurately represents real-world patients and therefore real-world effectiveness.

## Limitations

The data used in this study were retrospective and secondary. They were institutionally collected and processed in a structured, representative manner according to the Declaration of Helsinki. There is an increased interest in correct documentation since it directly affects hospital funding. However, these data did not provide any information on pre-hospital events, such as time-to-hospital or treatment prior to ECMO implantation, which are likely to affect survival. Additionally, the data did not provide information on the ingested drug or its pharmacokinetics and pharmacodynamics; only information on its classification was available. A propensity score matched control group was not feasible due to the unavailability of these informations. Due to the low usage of ECMO for cardiovascular medication poisoning, the analysis of the population is limited by a low case number. Furthermore, this study considers a significant time period, during which ECMO technology and clinical applications have significantly evolved. A detailed analysis of these changes over time was not possible within the scope of our data due to privacy constraints.

## Conclusion

This study demonstrates ECMO as a viable treatment option in cardiovascular medication poisoning, with a 63.3% survival rate. Although the patients were significantly older than in previous studies, the survival rate was higher. The frequent ingestion of multiple substances, often with suicidal intent, highlights the need for a comprehensive, interdisciplinary approach in managing such cases. The implementation of V-A ECMO for cardiovascular medication poisoning could potentially lead to higher survival rates. However, prospective randomised studies are necessary to further substantiate this approach.

## Data Availability

The Federal Statistical Office of Germany provided all data used in this study. These data were used under license for the current study and are not publicly available. The data are available from the corresponding author upon reasonable request and with permission from the Federal Statistical Office of Germany.
